# A Nature-Based Intervention for Promoting Physical Activity in Older Adults: A Qualitative Study Using the COM-B Model

**DOI:** 10.3390/ijerph21070843

**Published:** 2024-06-27

**Authors:** Katherine N. Irvine, Daniel Fisher, Margaret Currie, Kathryn Colley, Sara L. Warber

**Affiliations:** 1 Social, Economic, Geographical Sciences Department, James Hutton Institute, Aberdeen AB15 8QH, UK; dan.fisher@glasgow.ac.uk (D.F.); margaret.currie@hutton.ac.uk (M.C.); kathryn.colley@hutton.ac.uk (K.C.); 2 Centre for Public Policy, University of Glasgow, Glasgow G11 6EW, UK; 3 Department of Family Medicine, University of Michigan, Ann Arbor, MI 48104, USA; swarber@umich.edu; 4 NOVA Institute for Health, Baltimore, MD 21231, USA

**Keywords:** public health, prevention, non-pharmacological intervention, behaviour, physical health, mental health, green space, chronic health conditions

## Abstract

Physical inactivity contributes to over 800,000 deaths annually. Numerous non-pharmacological interventions provide a route to address this behavioural risk factor linked to the growth of non-communicable diseases. Here, we consider a nature-based intervention, specifically group outdoor health walks (GOHW), as a non-pharmacological intervention to increase physical activity and contribute to health and quality of life amongst older adults. We used the theoretically grounded Capability, Opportunity, Motivation, and Behaviour (COM-B) model as a lens to examine interviews with participants in a GOHW with an activity tracker and signposted by health clinics in Scotland, UK. Analysis identified capabilities, opportunities, and motivations, their impact on behaviour, and perceived physical and mental health. The application of the COM-B model to intervention evaluation allowed us to examine two separate behaviours, that of (i) engaging with the intervention itself, and (ii) incorporating the behaviour into one’s life that the intervention targets. Analysis identified emerging capabilities, opportunities, and motivations that supported additional health-promoting behaviours, including increased time outdoors in nature and leadership to self-organise continued group walks. We offer insight into the design of nature-based interventions to effectively engage older adults with chronic health conditions and foster personal behaviour change for health and well-being.

## 1. Introduction

Chronic noncommunicable diseases (NCDs) account for over 70% of deaths worldwide [1,2]. Governments in both the Global North and South are therefore faced with many pressing public health concerns associated with modern forms of living [3]; these include increases in chronic depression, obesity, cardiovascular disease, stroke, respiratory diseases, and diabetes, among others [2]. In Scotland, as in many countries, the population is ageing, a trend predicted to continue [4] with a concomitant increase in the prevalence of multiple health conditions [5]. Both preventive measures and treatments are needed to improve peoples’ health and quality of life while reducing the cost of healthcare demands [6,7].

Four modifiable behavioural risk factors have been linked with the growth of NCDs, namely: lack of physical activity, tobacco use, excessive alcohol consumption, and poor diet [8]. This paper addresses physical inactivity, which contributes to over 830,000 deaths annually [2]. Numerous non-pharmacologic interventions (NPIs) seek to promote physical activity [9,10], yet all face the challenge of promoting engagement with the intervention and determining whether the intervention affects the behaviour of interest. The behaviour change intervention model proposed by Michie et al. [11], with its focus on capabilities, opportunities, and motivations, and their impact on behaviour (COM-B), provides a useful lens for examining such challenges. We consider here the potential of nature-based interventions, specifically group outdoor health walks (GOHWs), to increase physical activity and contribute to health and quality of life amongst older adults. We use the COM-B model to examine interviews with participants in a GOHW combined with an activity tracker and signposted by health clinics.

### 1.1. Nature as an Intervention for Health and Well-Being

While the health benefits of physical activity are fairly well understood [12], linking physical activity to time spent outdoors may increase these benefits. Various systematic reviews have suggested causal links between time spent in nature and reduced levels of obesity [13], reduced cardiovascular mortality rates [14], and reduced levels of diabetes amongst older people [15]. For older adults, spending time in public green spaces has been found to lead to significantly lower anxiety and depression levels, and significantly higher relaxation and contentment levels [16]. Lab- and field-based experimental studies have demonstrated independent physiological and psychological effects of the outdoor environment over and above those of physical activity, with physical activity in a pleasant natural setting conferring greater benefit than in non-natural, built-up settings [17,18,19]. These effects can further promote healthy behaviours by boosting motivation to engage in physical activity and reducing perceptions of effort [20].

Policymakers are also aware of the benefits of spending time in green spaces. For example, in the UK, Public Health England’s [21] report highlights the need to improve access to green spaces for health reasons. Across Scotland, local councils are developing Green Space Networks aiming to protect and enhance green spaces within their boundaries for multiple benefits, including health (e.g., [22,23]. The use of green spaces is, however, unequal and varies by age, gender, cultural background, and socioeconomic circumstances [24,25,26]. Despite the aforementioned benefits of green space use, the capacity of older adults to engage in outdoor activities may be reduced with age and they may struggle to access outdoor natural settings [27,28].

Nature-based interventions (NBIs), which can be understood as activities or strategies to improve health and well-being through engaging with nature [3], are becoming increasingly accepted and have been prescribed by health departments [29]. Examples of NBIs include forest bathing [30,31], care-farming [32,33], wilderness therapy [34], green gyms [35,36], and outdoor exercise groups [37,38]. These NBIs can be either preventative or therapeutic depending on the health of the individuals involved. Another attractive feature of NBIs is that a single activity can provide multiple physical and psychosocial benefits (e.g., [35]). As a result, “when scaled up, [NBIs] could have significant and cost-effective implications for population health” [3] (p. 140).

Group outdoor health walks (GOHWs) are NBIs that are “short, safe, social, local, low level, led walk[s]” [39]. GOHWs can provide older adults with the opportunity to engage both with the outdoor environment and be active in a group setting. Specific benefits associated with organised walking groups include improved physical well-being (increased cardiovascular health; [40]), improved social well-being [28], as well as psychological and spiritual well-being [3,7,41].

### 1.2. COM-B Model as a Way to Understand NBI Behaviour Change

In general, NBIs aim to improve health and well-being outcomes for participants. Many will seek to do this by promoting behaviour change such as walking or taking part in other activities in a natural environment [3]. However, studies evaluating the effectiveness of NBIs demonstrate a great deal of heterogeneity in the outcomes measured [29,40], and behavioural change may be assumed rather than explicitly measured as an outcome. Recent reviews have highlighted the need to bring a behaviour change perspective to the study of NBIs [42,43].

**Figure 1:**
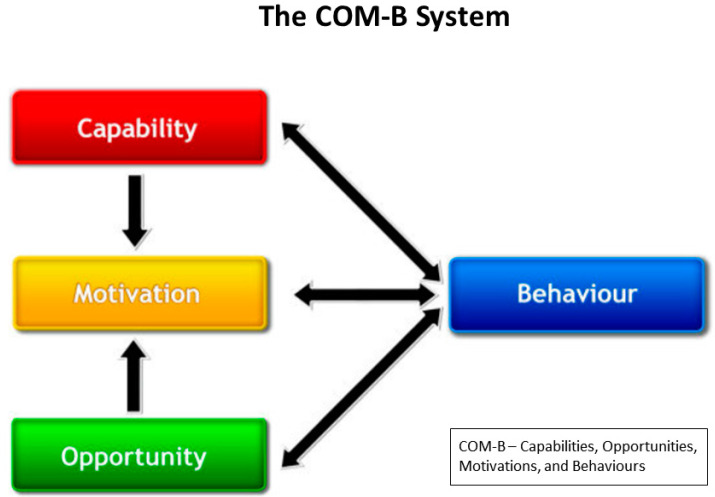
The COM-B model—a framework for understanding behaviour (reproduced from Michie et al. [11] under open license CC by 2.0).

Michie et al. [11] synthesised 19 different behaviour change models to develop an integrative framework for use in testing, evaluating, and improving interventions. At the core of this framework is the COM-B system (Figure 1) which recognises behaviour (B) as an outcome of the interaction between an individual’s capabilities (C), external opportunities (O), and an individual’s motivations (M). This system acknowledges the centrality of motivation, combining it with “the minimum number of additional factors needed to account for whether change in behaviour would occur” [11] (p. 4).

Capability can be understood as an individual’s physical and psychological ability to carry out an activity. Opportunity refers to features of the physical environment as well as determinants at a societal or community level that constrain or enable participation in a certain activity. Motivation includes both the conscious (e.g., goal-oriented planning) and subconscious (e.g., emotional, habitual) mental processes that influence behaviour. The COM-B model provides sufficient flexibility to allow for comparisons to be made across various interventions designed to affect people’s behaviours. While it is increasingly used to study many NPIs, it has yet to be applied to NBIs for adults, such as GOHWs.

### 1.3. Study Focus

One challenge with NPIs, including NBIs, is discovering ways of effectively engaging people in the intervention. Another significant challenge is knowing whether the intervention actually leads to incorporating the behaviour of interest into one’s life. Thus, a clearer understanding of the drivers for engaging in NPIs/NBIs and for changing behaviour would help identify worthwhile interventions and desirable improvements.

The application of existing behaviour models, such as the COM-B, to intervention evaluation allows us to examine two separate behaviours, that of (i) engaging with the intervention itself, and (ii) doing the behaviour that the intervention targets. The aim of this study was to qualitatively explore how the COM-B model can offer insight into the experience of older adults in a GOHW seeking to increase physical activity for health gain. The research questions that guided this study include:

1. How do antecedent capabilities, opportunities, and motivations (COMs) affect engaging in a GOHW?
2. How does programme engagement develop emergent COMs that affect ongoing physical activity?
3. How do these emergent COMs affect other health-promoting behaviours?
4. How does the chain of antecedent and emergent COM-Bs affect health and well-being among older adults with or at risk of NCDs?

## 2. Methods

### 2.1. Research Design

We adopted a qualitative approach using an instrumental case study design [44]. The research was conducted in rural Scotland (UK) and was designed to enable a group of older people to detail their experiences of taking part in a GOHW through their own stories. Using the lens of the COM-B model to examine older peoples’ capabilities, motivations, and external opportunities, we provide nuanced descriptions [45,46], along with interpretations [47], to promote understanding of older peoples’ behaviour change in relation to group health walks that take place outdoors.

### 2.2. Recruitment Process

There are currently two national parks located in Scotland (UK), one of which is the Cairngorms National Park. The Active Cairngorms Strategy, which promotes the use of the Park for physical activity ran a 12-week GOHW (from July to October 2017) monitoring participating individuals’ physical activity using an activity tracking system. The goals were to increase physical activity and to engage with local doctors’ surgeries to signpost patients to the walks. Participants were recruited to the activity tracker GOHW through posters on community notice boards and leaflets were placed at the doctors’ surgeries and distributed door to door. In total, eleven people completed the 12-week GOHW.

All participating walkers were made aware of this qualitative interview study by the walk leader. The walkers invited a project researcher (KNI) to join a walk and post-walk social time as a way of meeting potential participants and giving them further details of the research (such as the purpose of the study, intended use of data), as well as to experience a walk with them. For this study we sought to include all walkers who had taken part in the activity tracker GOWH, thus there were no exclusion criteria. Of the eleven potential participants, eight agreed to take part in the study. Reasons the other three potential participants did not agree to take part in the study related to ill-health. Another participant who joined the activity tracker GOHW part-way-through was also recruited for this study. Ethical approval was sought and granted by the James Hutton Institute’s Research Ethics Committee (119-2017).

### 2.3. Participants

The nine participants all lived in the same village located within the National Park. The age range of participants was from 50 to 80 years old. Three lived alone (two widowed, one single) and the remaining six were made up of three couples. Five participants were female and four were male. All were white British.

### 2.4. Data Collection

In April 2018, eight interviews were conducted (by KNI); seven of these were individual and one was paired (participants P6 and P7). During this time, the interviewer also participated in a second GOHW and the subsequent after-walk social time to gain further appreciation of the group and the geographic context. Interviews took place in a mutually agreed and quiet location. The interviewer reviewed the participants’ information sheets and consent forms, answered participants’ questions, and obtained written consent prior to commencing the interview. The semi-structured interview schedule consisted of open-ended questions with prompts that focused on how the GOHW had impacted their current behaviour. To break the ice, every participant’s interview began by asking them to share a story about the GOHW that was special to them. The schedule more specifically asked questions about recruitment and what motivated them to join, how they got involved, and whether there were any barriers to them joining. The next section explored their experience with and use of the activity tracker. Section 3 was focused on the locations of the walks and participants’ experiences, including being outside in nature. In the final set of questions, participants were asked to tell the interviewer anything else about the walking programme that was important to them and how the programme might have impacted their lives after the 12-week activity tracker programme had ended. Most interviews took around 50 min but ranged from 26 min to 80 min.

### 2.5. Analysis

The interviews were audio-recorded and anonymised prior to verbatim transcription. The interview transcripts were analysed through a process of in-depth thematic analysis [48]. NVivo (version 12) was used to create an initial coding frame based on the interview questions and existing GOHW literature (e.g., [7,41]. Further codes were added as they emerged in the analysis. We thus incorporated both a researcher-anticipated (etic) and participant-derived (emic) approach to data analysis [49]. The process began with the coding of one interview by two researchers (KNI, MC), the development of consensus, and the independent application of resultant codes to the remaining transcripts (KNI, MC, DF). In keeping with our public health interest in how interventions affect behaviour change such as the adoption of physical activity, we recoded the data using the lens of Michie et al.’s [11] COM-B model. We employed high-level codes of Capabilities, Opportunities, and Motivations (Table 1), and the data were then systematically analysed using the COM-B frame (KNI, SLW, DF).

### 2.6. Validity

We used several methods to ensure the validity and credibility of our analyses [50]. The first was having the interviewer spend time in the field with the participants by accompanying them on walks and to the after-walk social time on two separate occasions. This greater understanding of both the participants and the social and geographical context of the walking programme was brought to the coding discussions. We complemented this procedure with three researcher-based validity practices. First, we acknowledge our bias towards a public health point of view in this particular analysis. We expressly chose an existing behaviour change model, the COM-B, and applied it to our data. As we did this, we continuously sought both confirming and disconfirming evidence from the interview data. Finally, throughout our iterative analysis, we employed triangulation through discussion and consensus building among four researchers from differing disciplines: environmental psychology, medicine, and geography.

## 3. Results

**Figure 2:**
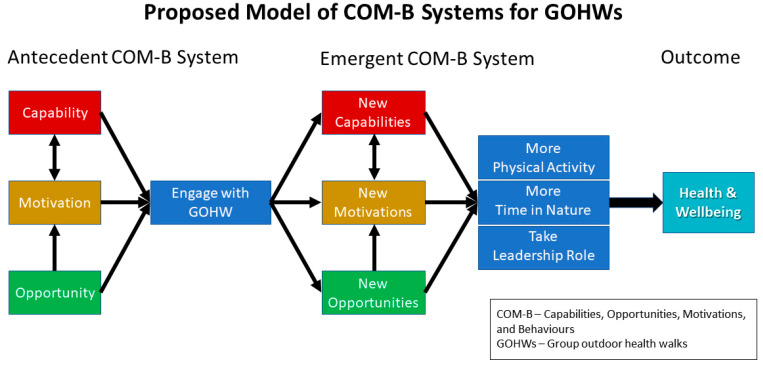
Arrows illustrate relationships within and between antecedent and emerging COM-B systems. GOHW stands for Group Outdoor Health Walk. (Diagram created by SLW and KNI; informed by [11,51]).

We structured findings by the components of the COM-B system with an additional section identifying associated health and well-being experiences. In discussing aspects of participants’ capabilities (C), opportunities (O), and motivation (M), we looked at both those that were present to bring individuals into the GOHW (antecedent) as well as how these changed or new ones emerged (emergent) through experience with the GOHW programme. Figure 2 provides a visual representation of this antecedent and emergent nature of the COM-B model amongst the individual elements to inform emergent behaviour as well as health and well-being.

### 3.1. Physical Capabilities

The capacity to physically engage in a given behaviour refers to the physical skills, strength, and stamina of the individual. In this case, participating in GOHWs requires some level of strength, stamina, and ability to walk.

#### 3.1.1. Physical Capabilities—Antecedent

When describing their physical capabilities before joining the GOHW, participants spoke about their lack of fitness, advancing age, decreased strength, and/or health setbacks that limited being physically active. One participant described others in the group as either “walkers”, or “one or two who are not”, and a lot who were “real hardened walkers that have been to the top of the hills in their young days but are just not fit anymore” (P1). Another confirmed, “I used to be a long distance walker anyway. I used to do up to 25 miles …, but I’m getting a bit old for that now” (P5). Another admitted, “my body is so decrepit anyway. That was another reason for going on the group, to strengthen my legs more than anything else” (P2).

Some had health issues that limited their physical abilities prior to joining the walks. Such health issues included participants who had experienced a series of falls and others who were recovering from operations. Summing up their collective previous physical capabilities, one participant remembered a recent walk with a steep incline, “I think if we’d done that walk at the beginning …, thinking back…, I think there would have been quite a few saying, ‘I’ve had enough’” (P3).

#### 3.1.2. Physical Capabilities—Emergent

As participants progressed through the weeks with the outdoor group walks, their changing physical capabilities became evident. One participant observed:

The chap across there, he used to walk to the letterbox here and back. He’s never walked any further than that. Since the walk, he’s been walking two miles. … [and] I got to the stage where I thought, ‘This is really good, I’m glad we did this because it’s made a big difference’… I walk more now than I did. (P4)

Changed physical ability is demonstrated in another participant’s comment, who noted that “when we were going for walks together we were finding, ‘hey, look, that has helped’. We can actually push ourselves a bit more” (P3). Another walker (P2) described herself initially as “at the tail end” of the group “puffing away” and “wondering at that stage whether I had actually done the right thing”. She subsequently observed that, over time, “as we all became slightly stronger, we’d go further afield”.

This same participant (P2) reflected on another walker’s changed physical ability: “She’s got a breathing problem, but even she was astounded that … She walked the whole length without stopping once and she was just over the moon that she’d managed to do that”. These observations were echoed by another walker who observed “the three miles doesn’t seem like three miles now” (P9). Collectively, these comments are suggestive of how group members’ physical capabilities had improved significantly during the course of the repeated GOHWs.

### 3.2. Psychological Capabilities

This aspect of capabilities focuses on participants’ psychological capacity to engage in joining a walking group in order to increase physical activity. Psychological capacity might include knowing that a behaviour needs to be modified and why, what is involved in making these changes, or how to do the behaviour. For this GOHW, there was awareness of the need for physical activity yet a lack of awareness of places to walk. Over time, participants gained more knowledge about walk location, how to navigate being physically active outdoors, and developed more understanding of their own physical capacities.

#### 3.2.1. Psychological Capabilities—Antecedent

Several participants spoke of being aware of the need to be more physically active. For example, one walker highlighted this in their observations about the GOHW: “I said [to a friend] that I felt [the walking group] would be quite good; anything that wasn’t too strenuous to start off with I would be interested in….” (P2).

Additional insight into participants’ pre-walk knowledge comes through comments such as “quite a number of our group had never been outside the village…in a car, yes, but not walking” (P5). This suggests a lack of knowledge about areas for walking other than within the village where participants lived. One participant spoke of this in terms of how a lack of familiarity and comfort might influence one’s willingness to go out walking in a more natural area:

…some of the people are happier to walk around the village because they don’t have to confront that element of if there was nothing around about they were familiar with… I feel comfortable in that [natural] environment but if you haven’t done that before, its not part of your comfort zone, and we’ve all got comfort zones. (P8)

#### 3.2.2. Psychological Capabilities—Emergent

Participation in the GOHW developed psychological capabilities in several ways. First, there occurred an “expanding [of] our knowledge within the area…and [an] opening [of] one’s mind” (P3). Comments about the walks, such as “we’ve never been down here before” (P4), illustrate this growing awareness of places for walking in the local area. This same participant described how they would “try to go and maybe do the reverse or…cut through a different way” (P4), demonstrating a growing sense of mental flexibility in their understanding of how the walks could be utilised to support increased physical activity. Participants would also ask one another to suggest walking routes that the group could try, such that an informal sharing of information developed between the walkers. As a result, participants were building an understanding of effective ways to engage in being more physically active through outdoor walking. This expansion of knowledge was not only about where to go for a walk but also about the natural environment, such as the difference between a male and female holly tree.

A second psychological capability emerging from GOHW participation pertains to being able to navigate one’s way around outdoors, assess the terrain and length of the walk for difficulty, and be prepared for the elements. Participants often spoke of these in light of other’s abilities. For example, one participant described how the length of a walk could be reduced; “if some people wanted to go shorter, there’s a little picnic area that you can just…go to the picnic area and come back” (P1). The importance of having the knowledge and skills about spending time outdoors was also highlighted in relation to changeable weather. One individual described this in terms of:

I would be one that respects the nature that we’re in. While [Scotland] is a beautiful country… [the weather] can also turn.… there is nothing worse than thinking, ‘Oh, it’s a lovely day, I don’t need a coat today’, and you’re half an hour from anywhere and all of a sudden you get hit by something. (P3)

One participant commented that venturing beyond areas that were familiar might be difficult for some, observing that “some people could not find north or south without a compass to tell them” (P8). This hints at the importance of building knowledge and skills associated with finding one’s way around the environment or at least going with someone who does have this capability (“as long as they’ve got somebody to point them in the direction” [P8]).

A third psychological capability emerging from the interviews was about developing greater awareness and knowledge of physical activity and how this relates to one’s own body. This relationship was understood in terms of both need and ability. One participant spoke of reaching a point of “know[ing] if I’m not doing enough exercise, so I get up and go” (P9). Regarding ability, one individual noted that they were now able to “get a mental reference to approximat[e] how far walks are. [For example] just doing round the field close to me won’t be enough [for 2000 steps] but perhaps going in the woods and coming out back around in a circle will be” (P3). These comments point toward the emergent knowledge and skill for assessing one’s physical activity levels and how one might be able to increase it. In both instances, this growing psychological capability fosters engagement in the behaviour of increasing one’s physical activity.

### 3.3. Physical Opportunity

This element considers factors within the external environment that support or restrict engagement in the targeted behaviour. Key physical opportunities that were supportive of behaviour encompassed in the GOHW include the use of an activity tracker, level terrain, good weather, pathways available, the environment around the pathways, and cars to access some pathways. There were a few mentions of physical barriers that could hold walkers back or had to be surmounted, including difficult terrain, bad weather, and risk from cars or bicyclists.

#### 3.3.1. Physical Opportunities-Antecedent

The physical geography of the participants’ local areas was an important aspect of physical opportunity for engaging new walkers. These walkers tended to start with walks around the village that were flat and closer to home.

A unique part of this GOHW was that participants were given an activity tracker, providing a new physical opportunity for measuring and prompting physical activity. As one person acknowledged, “...the little thing… was handy. It shows you how many steps you’d do and you can increase it to your own fitness” (P5).

#### 3.3.2. Physical Opportunities—Emergent

As the weeks passed, group members encountered physical opportunities that supported their personal physical activity and/or that supported or hindered more challenging group walks. Weather, for example, could be a physical reality that interfered, whether rain or sudden snow, or an opportunity, if the weather was better. One walker commented, “We meet up and if the weather’s atrocious we just have a coffee.… Particularly during the winter” (P3). Another walker had reached the level of getting out for a walk every day, but not in the rain:

Most days, yes [I go for a walk]. I mean, if it’s pouring with rain, I don’t go. I’m not *that* enthusiastic (laughing). No. If I get caught out in the rain, that’s a different ball game. (P5)

As the walkers ventured farther afield on their own, they encountered other physical barriers, such as cars and bicycles. One participant commented “...providing it’s [the walk] not in the street; cars don’t exactly [slow down]” (P9). This same individual expanded further, noting that “These bicycles, they don’t have bells or anything, and they come up behind you and you don’t hear them. So, you have to be aware of that”.

After becoming involved in the walking group, the physical geography of local areas was an important physical opportunity for promoting further physical activity and getting outdoors either alone or with the group. Mostly flat, well-maintained pathways with appropriate rest stops were key for adding walks to the repertoire of options each week. One person identified a new physical opportunity for the walk group because of a newly built path:

They just built a new path. Before, it was very treacherous… It was slippery sometimes and muddy but since they put the new thing in, we’ve done that twice now. (P4)

With time, the group took on more challenging terrain:

I think that [route] took us over more different terrains as well which were … difficult walking: with large stones that you’re walking on that were moving, so you thought, ‘yes, okay [I can do that]’. (P3)

The extended quote below gives a good picture of the characteristics of pathways and spaces for walking that make them a good physical opportunity for the whole group:

…there are possibilities for everybody to be catered for and do slightly different walks. … [Another place is] open and it’s straight and it’s long, and sometimes there is a circular bit you can do, but if we are not feeling as fit as that we’ll go a bit and go back the same way and get back to the car, but it needs a car. (P9)

This comment from P9 also points out that these physical opportunities often require another physical opportunity: a car to get to the place. This statement contrasts with the village walks that are available all the time.

Part of the available physical opportunities includes the particular environment in which one is walking, as emphasized in these quotes:

You get the combination of being off the road where it’s quiet in the trees, a fair bit of natural history around about you whether it be birds or bees or something like that, and scenery changing. That’s what I like about walking. (P8)

… the first time you go somewhere like [Redacted place name] and you see the dolphins for the first time, you’re seeing nature at its absolute best. We’re lucky around here that we could go to … a nice little walk at the top of the loch where the car park is. We could do that and you can see loads of different things, particularly deer at the right time of year. (P3)

The physical opportunities of activity trackers, good weather, safe paths with variety, and interesting environments were key to bringing people into the walking group and keeping them coming back. Many clearly also availed themselves of these physical opportunities to undertake more physical activity outdoors on their own.

### 3.4. Social Opportunity

Social opportunity is how the cultural milieu made up of interpersonal influences, social cues, and cultural norms influences our thinking and ultimately our behaviours. Our participants provide narratives that emphasise how interpersonal influence, changing social norms, and peer support prompted them to join a walking group and stay with the programme.

#### 3.4.1. Social Opportunity—Antecedent

Numerous participants highlighted how interpersonal influence played a role in starting people walking or in bringing new walkers into the group. Personal influence via a direct social invitation made a difference for some as illustrated in the following examples:

…she came herself initially and then I saw her in the village one day… [with her husband who won’t go anywhere], so I was blethering away… and I said, ‘Oh, hello. Why are you not walking then?’. The next Friday, he came and he’s come back! (P1)

… so I said to her, ‘Don’t sit at home and get depressed, get out and come with us, come to the group’. I didn’t think she would but the next week, she was there and she’s stayed ever since. (P4)

The second powerful social opportunity was represented by the involvement of the local medical General Practitioners’ (GP) office (referred to as a surgery in the UK). In this case the existing social norm of the National Health Service (NHS) which attends to people’s health and a word from someone, e.g., “I think the GPs were encouraging [the GOHW] with certain people” (P1), fostered engagement. A new poster at the surgery advertising the programme further supported a social norm of engaging with the GOHW.

The social norming of a poster could also backfire by choosing a limiting image, as noted by this participant: “[The poster] was in the doctors’ surgery but we didn’t really take much notice of it because the photo … looked like old people and we think we’re not that old” (P4). However, for this individual, a more powerful purveyor of social norms, the TV, had whetted their appetite for a walking group such that when one was set up in the area, they were ready to join:

[The walking group] was just something I was waiting to happen because I’d seen it on the TV and I thought… ‘Oh, I wish that would happen here’, and it did and I thought, ‘Right, we’re in there’. (P4)

For others, it was the combination of interpersonal influence and social influence from the GP’s office as well as the National Park:

Why did I get involved? Well, I was introduced to it by … one of the other members here. … there were another couple there as well. They were from [the] National Health [Service] and the Cairngorm National Park. So, we had a chat with them and they asked us if we’d like to join. I said, ‘Well, why not?’ … My wife and I both decided to join the group. (P5)

#### 3.4.2. Social Opportunity—Emergent

Behaviour change is difficult to initiate and challenging to sustain. Walkers provided insight into how new social norms overcame this common constraint: “put it this way, perhaps if [Redacted participant’s name] hadn’t been going I could have quite easily [thought]… ‘Well, I won’t bother after all’” (P3). The group as a whole was also important:

I saw the social side actually helped you to actually do it and motivated you into actually doing it. We’d both been ill for the last couple of months…so you almost feel as if you need to go back and say ‘hello’ and get back into it. (P7)

Even in the case of an especially challenging walk, the presence of social norms helped this walker carry on:

I would have turned back, I would have definitely turned back but because there were other people there… I wasn’t even encouraged, I just wanted to because I didn’t want them to think, ‘I knew she couldn’t do it’. (P7)

Social opportunities played a key role in bringing people into the GOHW, including the interpersonal influence of direct invitations to join and social norming provided by the local medical practice, the national park, and TV advertising. The new social group of walkers that emerged provided a new social milieu and peer support that helped overcome barriers of previous habits and limitations.

### 3.5. Reflective Motivation

Reflective motivation considers the conscious mental processes that can influence behaviour. These conscious processes might include goal setting, planning, analytic thought, decision-making, and evaluations or beliefs about the benefits or drawbacks associated with a particular behaviour. This dimension of motivation emphasises the choice and intention behind engaging in outdoor walking as a behaviour to increase physical activity. In this GOHW, we see examples of incentives and goal setting, beliefs about group walking as a way to increase physical activity, and alignment with one’s identity and interests.

#### 3.5.1. Reflective Motivation—Antecedent

The fact that this GOHW included an activity tracker was evaluated as a benefit that stimulated the decision to join this particular walking group. For example:

Yes, the tracker seemed to be an incentive because if you were speaking to other people in the village that weren’t on the walking, they knew about it and they would say, ‘Oh, that’s the Friday walk and you get a fancy tracker’, and you think, ‘Yes, that’s right, that’s the one’ (laughing). So, this tracker was obviously an incentive. (P1)

Another way the activity tracker drove participation in the walking programme was “that somebody was tracking us for the 12 weeks just to see the difference between when we started and how it motivated us to the end” (P4). This comment suggests a conscious decision to join the walk in light of the goal-setting incentive to monitor progress. It additionally exemplifies a belief that their behaviour can successfully be modified. Other comments by this same individual suggest that taking part in a GOHW to increase physical activity was aligned with their beliefs that being outside is good for you. For example: “I’m an outside person anyway. I just love it, it’s great. It’s really good” (P4). For those who thought of themselves as an outdoor person, outdoor walking was considered an activity compatible with their sense of identity.

#### 3.5.2. Reflective Motivation—Emergent

The weekly walks meant that there “was a purpose for a Friday” (P4). This is suggestive of the importance of choice and a purposefully organised shape to the week to support the commitment to physical activity.

One participant reflected on the way in which the activity tracker played into walkers’ beliefs and fostered an action plan for achieving step count goals. They observed: “Well, it was setting goals. That was the main thing, setting goals for your own fitness. Some had a bit of a competition with others as well…so yes, a wee bit of competition was always good for everything” (P5). A dialogue between two participants (P6/P7) highlighted the way in which the weekly personal check-in supported a belief in one’s ability to engage successfully in increasing one’s physical activity:

P6: A target.

P7: No, there was no pressure.

P6: It was just in the comparison, the [walk leader] would look at [the activity tracker] and say ‘Oh, you’ve done really well this week, so many steps more’.

Participants also believed that being part of the GOHW had been good for them. One walker described this in relation to the effect on others, observing that “it’s been a very valuable thing for quite a few people in very different ways…it’s giving them a boost” (P9). Greater specificity is provided by the following walker who observed the increased confidence they now had in their walking-related leadership behaviour:

It’s been good for me as well because I don’t get involved in many things in the village. I don’t feel as though I’m confident enough. …it’s given me that bit to stand up and say, ‘Here I am, and this is what we’re going to do’…which has been good for me really. (P4)

Another noted the opportunity the GOHW gave them to see what was going on in the area: “I wanted to see the new path that they had put in…there was an element of interest” (P8). This is suggestive of an additional layer of intentionality behind engaging in walking with the GOHW.

### 3.6. Automatic Motivation

In this section, we consider the non-conscious processes behind joining the GOHW and being physically active. These include emotional reactions, for example, those that might facilitate or act as a barrier to engaging in or those that might arise from having done the behaviour. It also considers the habits, desires (such as urges or cravings) or inhibitions that might foster or hinder engagement in a behaviour. Here, we see the role habitual patterns, impulses, and various emotions played in both the joining and the physical activity behaviour.

#### 3.6.1. Automatic Motivation—Antecedent

Participants noted the role that habit played in their lack of physical activity prior to joining the walking group. One individual described this as “I got into a bit of a rut, a bad spell…of not walking and I just stuck to that” (P6). Another explained that, prior to joining, “Fridays [the GOHW day] just came and went and every day just came and went” noting that “we didn’t do much at all” (P4).

Individuals also spoke of automatic processes involving emotions and impulses. The former is illustrated by a participant noting the “feeling of security…it’s reassuring if you’ve got somebody with you” in case there is “a bit of an upset and…collapse” (P8). Illustrative of an impulsive motivation that influenced joining the group is the observation a participant made about the provided activity tracker: “a lot of people probably joined for that reason, maybe [thinking], ‘oh you get free this and free that’” (P4).

#### 3.6.2. Automatic Motivation—Emergent

Feeling states that were emerging amongst participants include a sense of pleasure associated with “the weather improving, …finding different walks, and obviously the scenery that goes with it” (P3), as well as amazement. This latter, mentioned by a participant who “used to get very angry” because of their physical limitations, is described as follows: “It was just amazing that I could get out there and do it and not feel hemmed in the house” (P2).

### 3.7. Behaviours

Joining the walking programme was the initial behaviour in which participants needed to engage. We anticipated that involvement in the GOHW would lead to more group and independent physical activity, as well as more time outdoors. We also anticipated socially focused behaviour (explored in depth in [28]. An unanticipated behaviour, that of becoming a walk leader, was also identified. Here we consider these three emerging behaviours: physical activity, spending time outdoors, and leadership.

Within the participant’s comments was a clear sense that being involved in the GOHW was increasing their physical activity beyond the walking group activity in several ways. One individual noted that they are being more physically active at home: “at the beginning…I used to say ‘could you go up the stairs for this? Could you go up the stairs for that?’ Whereas now…. I just get on and do it” (P2). This same individual continued:

I do see such a big difference. I walk to the doctors now instead of taking the car. I walk to the shops, whereas before it would be the car. …I would say ‘Let’s walk into the village and get the shopping’. (P2)

Another individual in the group noted, “[before] we didn’t do much at all. We [would] go walking but not as much as we do now…” (P4). They further reflected that their partner “wouldn’t go out in the afternoons. He would just sit but it’s been good and he [now] just goes [for a walk] by himself” (P4).

Several of the comments explicitly make clear that the walking behaviour is occurring outdoors. One individual described this in relation to the group as follows: “That’s the main thing, getting us out” (P5). In a subsequent comment, this same walker provided further detail as to the location of the outdoor walks they were now taking: “We sometimes get out of the village, … up to [local natural areas]” (P5). Another walker noted that they were now able to plan holidays in places that require more walking to local amenities than they had been able to previously.

With regard to becoming a volunteer walk leader, five individuals in the group had or were planning to take the GOHW leadership training. Several of these spoke about how involvement as a walker had generated an interest in leadership. For example, one individual realised during their reflections on the effort that had gone into organising the GOHW that there needed to be new leaders to keep it going, commenting: “Right, this is not going to stop, we can’t stop here…why should we stop? We’ve got to carry on” (P4). Another spoke of how their experience as a participant in the GOHW had illustrated the myriad roles played by the leader, noting that although “I had no intention in becoming a leader…I’ll go on the course…and I might be able to help at the rear if I know what it’s all about” (P6). They further commented, “Even if you’re not going to do any of those things, I still think there’s benefit in actually going on that training…gaining insights into what the lead is supposed to be doing really so [you can be supportive]” (P6).

Participants identified several behaviours within the walk leader role that they were now undertaking. These focused largely on risk assessment and being a motivator. The former focused on path selection and safety on the day. Participants noted the importance of “at the start of the walk…if there’s anybody new that if they feel unwell or finding it too hard, they voice it so we can rearrange things” to accommodate (P1). This same risk assessment element continued throughout the walk to “take it at a pace that I thought people would be comfortable at” (P3), to integrate rests into the walks, and to keep the group together.

A second aspect of leadership behaviour identified by participants is that of playing the role of motivator for others. One way in which this was done was to keep track of “how far we’ve walked…to see how adventurous we were” or to remind walkers “you need your steps” (P4). Another was to find ways to keep walkers interested in walking, for example, by asking walkers which walk they would like to go on (“Where do you fancy going walking today?” [P1]).

These three emerging behaviours—personal physical activity, being outdoors in nature, and leadership—illustrate the influence of the GOHW on expanding capabilities, opportunities, and motivations. These new behaviours can, in turn, support changes in health and well-being.

### 3.8. Health and Well-Being

Group outdoor health walks have an overall objective of improving health and well-being. The COM-B model of understanding behaviour change also intends to elucidate interventions that improve health. In this section, we look at participants’ views of how health was safeguarded, their health/well-being issues, and their perceptions of health/well-being improvements.

#### 3.8.1. Safeguarding Health

Participants were required to identify their health issues to the walk leaders; however, that information was not shared with other group members. In addition, the group setting provided a sense of safety and security if something untoward were to happen. “It’s reassuring if you’ve got somebody with you who can get you home or to the doctor or call the ambulance… There’s a feeling of security in that, I think” (P8). Overall, health and safety were considered in the basic setup of the group health walks. This is especially important in light of the actual health of the participants.

#### 3.8.2. Health and Well-Being Issues

Thinking about the health of the group, one person summed it up with, “We all had disabilities of some description” (P2). Another agreed that the walks were for everybody, “and it doesn’t matter whether it’s cardiac or whether you’re recovering from a hip operation or replacement, or a stroke or anything like that” (P8). In fact, only one of the interviewees did not share any self-identified health problems. Most identified physical problems, some had mental/emotional challenges, and some mentioned a desire for social connections. Here we will focus on physical and mental well-being. How GOHWs facilitate social well-being has been explored in a previous paper [28].

Pre-existing physical health issues included falls and balance issues, pain in the joints or back, past hip operations, previous heart attacks or surgery, strokes, chronic lung or neurologic diseases, hearing loss, and systemic infection. Mental well-being issues spanned from difficulty with concentration to being ‘down in the dumps’ to severe depression. Some of these mental/emotional issues were associated with chronic physical distress, while some were the main health reasons for joining the group.

#### 3.8.3. Health and Well-Being Improvements

People in the group lost weight and got stronger; they felt happy and cheerful on the walks and looked forward to walk days. One said, “I think I’ve only fallen once [since joining the GOHW] … I now can see beyond [the pain]” and “it’s changed my view of life and I’m not so angry anymore. I’ve learnt to come to terms with my body and its aches and pain” (P2). Another countered, “I wouldn’t say it’s impacted my life, but it has done a lot of good, and it’s made me more aware that I should be out getting exercise” (P7). One person said about their spouse: “He looks much healthier… It’s made a big difference to his health. Also, he’s just different, he’s out and about and not waiting for me all the time to do things” (P4).

While no one was claiming that their very serious health issues were cured, they were seeing a change in themselves or their loved ones. Stronger, lighter weight, exercising, fewer falls, happier, and able to cope with pain; these contribute to a healthier lifestyle, better mental well-being, and improved quality of life. For older people with chronic health conditions, this adds up to a healthier life.

## 4. Discussion

This paper reports findings from a qualitative case study exploration of the drivers for engaging in and adopting new health behaviours through involvement in a nature-based intervention (NBI), specifically a group outdoor health walk (GOHW) in Scotland, UK. Using the analytical lens of the established COM-B model of behaviour [11], interviews with older adults with chronic health conditions revealed a set of capabilities, opportunities, and motivations that acted to support or hinder initial engagement. We additionally identified the emergence of new capabilities, opportunities, and motivations that influenced ongoing physical activity, time spent outdoors in nature, and leadership behaviours. Our participants further drew links between the GOHW experience and their own health and well-being. Findings contribute knowledge of drivers behind two important yet distinct behavioural challenges of NBIs—joining the intervention and integrating the targeted behaviour into one’s life. In this way, our study responds to calls to incorporate a behaviour change perspective—and in particular the COM-B model—for research into the use of NBIs for health behaviour change [42,43]. To the best of our knowledge, this is the first application of the COM-B to an NBI for adults and the first framing of NBIs as a subset of NPIs.

### 4.1. Summary of Findings

In this section, we structure our discussion of findings by our four research questions. In situating these within the physical activity literature, we primarily consider qualitative studies that also utilise the COM-B model to examine physical activity NPIs for adults. We further call on literature from environmental psychology and outdoor recreation as we consider the importance of exercising in nature.

#### 4.1.1. How Do Antecedent (Existing) Capabilities, Opportunities, and Motivations (COMs) Affect Engaging in a GOHW?

**Figure 3:**
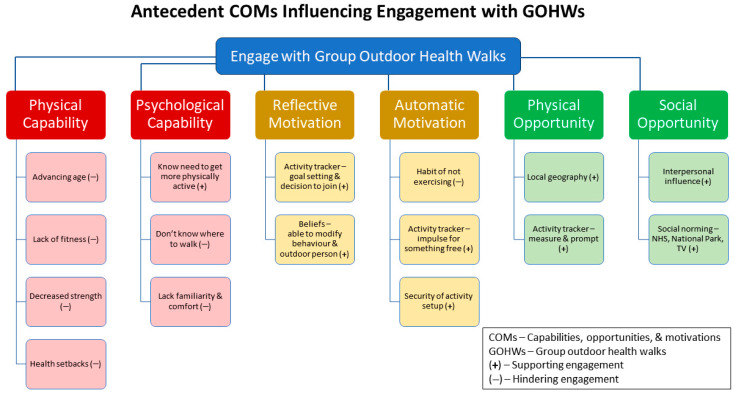
Antecedent COMs that influence the behaviour of engaging with group outdoor health walks. (+) stands for facilitating COMs; (−) stands for inhibiting COMs. (Diagram created by SLW and KNI).

Pre-existing COMs could either tend to inhibit engagement with the GOHW or promote it (Figure 3). On balance, the positive influences outweighed the negative for these walk members. Poor physical capabilities, like age, health, or lack of fitness, were likely to hold people back. This has been a well-documented issue for older adults in relation to engaging in physical activity [52] and is an identified constraint when considering undertaking physical activity in an outdoor setting [53]. Likewise, some psychological capabilities were drawbacks, such as not knowing where to walk or feeling uncomfortable beyond their immediate environs. However, importantly, most participants were aware that they needed to be more active, a positive psychological capability. The presence of such knowledge was also identified by Barrett et al. [54] in their COM-B-informed study of a non-group physical activity coaching intervention. Here we illustrate the importance of recognising this existing psychological capability for individuals targeted for GOHWs.

Physical opportunities, such as locally flat geography and the prospect of an activity tracker to measure and prompt exercise, weighed in positively. Social opportunities for engaging in the GOHW were strongly positive, including interpersonal influence. Our previous research on GOHWs identified anticipated social engagement as an additional driver for joining [28]. A key social opportunity was the social norming of support and advertisement from the local NHS, a walking programme organised by the National Park, and advertising on TV. Social opportunities were a significant factor for women of lower socioeconomic status (average age 53; range 30–69) in joining an outdoor exercise programme [55], and the social norming of a physical activity prescription was significant in a Swedish study [56]. Both of these studies also utilised the COM-B lens for analysis; the latter primarily focused on older adults. In a recent systematic review of qualitative studies of older adults (57+) and physical activity interventions, Meredith et al. [52] identified socio-cultural discourses and messaging in the media as important social opportunity factors influencing older adults’ involvement in physical activity.

These positive capabilities and opportunities combined with reflective motivations to encourage joining the GOHW. These motivations included a belief in the ability to change, an identity as an outdoors person, and an interest in goal setting. Having a conviction that physical activity is a good thing to do was also an identified facilitating factor in Meredith et al.’s review [52] and was a noted motivator in two studies of physical activity NPIs with distance motivational coaching for adults (30+ years old) [54,56]. Irvine et al. [41] in their study of GOHW with older adult participants (range 63–81 years old) identified activity trackers as a strong motivator for joining. Automatic motivations of feeling secure in the design of the GOHW and the impulsive desire for the free tracker also contributed to overcoming walkers’ discomforts, physical incapacities, and the powerful negative automatic motivation of habitually not walking. These motivations are not well described in other studies of physical activity using the COM-B model with qualitative data. Meredith et al. [52], however, also categorised physical activity habits as automatic motivations.

#### 4.1.2. How Does Programme Engagement Create Emergent COMs That Affect Physical Activity?

**Figure 4:**
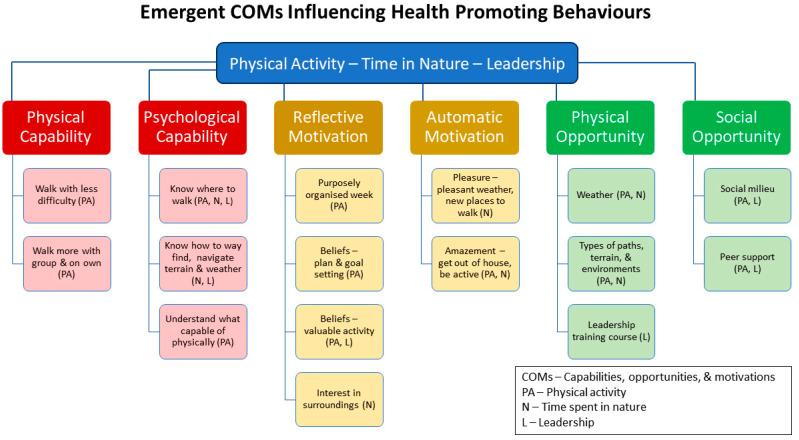
Emerging COMs that influence the health-promoting behaviours of physical activity (PA), time spent in nature (N), and walking group leadership (L). (Diagram created by SLW and KNI).

The emergent capabilities and motivations stemming from being part of the GOWH increased this group of walkers’ physical activity (Figure 4). The change in physical capability supported walking with less difficulty and walking more. Walkers spoke of both regularly attending the weekly group walks and integrating more walking into their daily life through, for example, errands or trips to the doctor. While other qualitative studies of group-based NPIs targeting physical activity often identify changes in this behaviour, few differentiate between activity within and activity outwith the programme (e.g., [55], a contribution of our findings. Our previous qualitative and mixed-method study of GOHWs with an activity tracker also identified an increase in physical activity beyond the programme [41]. The emerging understanding of what one is physically capable of and the increased knowledge about locations for walks exemplify the expanding psychological capabilities that facilitated being more physically active. Andersen et al.’s [56] study of a counsellor-supported physical activity prescription also noted the role that awareness of one’s own physical capacities plays in supporting increased physical activity. Purposefully organising one’s week and setting goals alongside growing beliefs that walking was a valuable activity illustrate emergent reflective motivations, insight mirrored in Barrett et al.’s [54] interviews of adults enrolled in an individualised NPI. Coupled with these more conscious mental processes were feelings (an automatic motivation) of amazement at being able to be physically active. McKeon et al. [55] also identified the importance of feelings (i.e., enjoyment) as a facilitator for engaging in physical activity behaviour.

Being part of the GOHW brought individuals into contact with a new set of external physical and social opportunities that supported physical activity behaviour. Well-maintained, relatively flat paths with places to rest and that went through interesting and varied environments facilitated walkers getting out and going further. This ‘fit’ between the physical environment and one’s behavioural goal—environmental affordance [57]—has been identified in other qualitative studies of physical activity NPIs [52]. Time during the day was now seen as available for walking and thus increasing physical activity; others have identified the link between time and the reflective motivation of valuing physical activity [54]. A physical opportunity, weather, could promote physical activity through outdoor walking if pleasant or inhibit if cold, wet, or snowy. Weather is seen as either positive or negative in numerous studies of outdoor physical activity [52,53,55,58]. The group structure of the walking programme provided a new social opportunity for peer support that facilitated sustained engagement with walking. Individuals were now embedded within a new social milieu, one in which other people in their lives were also seeking to improve their health through physical activity. The contribution of social opportunities derived from group NPIs toward supporting physical activity is broadly supported in the qualitative literature [28,55,59]; for review see [52].

#### 4.1.3. How Do These Emergent COMs Affect Other Health-Promoting Behaviours?

Spending time outdoors in nature was an additional health-promoting behaviour influenced by the emerging COMs (Figure 4). Here, the emerging physical strength and stamina (physical capability) facilitated walking further afield on paths that took the walkers through more varied natural settings, allowing them to further their engagement with nature. This was evident for the group walks but also outwith, for example, planning holiday accommodations that were a further walk to local amenities. Walkers also (re)-learned about how to navigate being physically active outdoors, such as finding one’s way around, traversing different types of terrain, and managing changing weather (psychological capability). An important aspect of people–environment interactions is the availability of a mental model or internal representation of an external environment that can guide people’s perceptions, decisions, and behaviour, such as wayfinding [60,61]. Here we see that taking part in a structured GOHW can support the expansion of one’s mental map of the outdoor environment—its paths, terrain, and weather—thereby increasing psychological capability for engagement in outdoor physical activity.

Emerging motivations that supported increased time spent in nature included intentional interest in exploring the surrounding area (reflective motivation) and pleasant feelings (automatic motivation) evoked by natural scenery and seasonal changes. While considerable research identifies the positive emotions derived from time spent in nature, including through GOHWs (e.g., [7]), identification of this as a factor that supports additional engagement in outdoor behaviour is a contribution to understanding NBIs for physical activity. While McKeon et al. [55] identified enjoyment as a motivating factor, this was largely associated with varied physical activity options and the social dimension; here we identify the emotion associated with nature itself as a driver for engaging in outdoor behaviour.

Physical opportunities additionally played an important role in supporting outdoor behaviour. The availability of newly repaved paths that had previously been difficult to traverse made it possible for the walkers to reach countryside areas. Paths that were circular or could be shortened, depending on individual needs, also made it possible for the group to venture out of the village into more natural places. Access to outdoor spaces, for which paths play an important role, is a commonly cited constraint to spending time in nature, particularly for older people [53]; our findings lend further support for the necessity of appropriate physical opportunities for outdoor behaviour.

Some individuals displayed an unanticipated emergent behaviour by stepping into leadership to self-organise the continuing availability of group walks (Figure 4). The belief that being part of the walking group had benefited themselves and others, and increased confidence in interacting with others, motivated several walkers to undertake training in how to lead the group (reflective motivation). This motivation was supported by the availability of walk leader training resources (physical opportunity) offered by the organisation that set up the original group. Engaging in leadership behaviour was also influenced by the fact that others in the group were undertaking the leadership training, which offered peer support (social opportunity). Lastly, the learning that had gone on while being a participant in the originally organised GOHW—about paths, safety, and how to be supportive of others—brought an expanded psychological capability to their decision to become a walk leader.

Peer-delivered interventions can effectively promote change in an individual’s physical activity [62], particularly among older adults [63]. This is a model used for GOHWs in Scotland [64]. Recruitment of volunteer peer leaders can present challenges [65]. Our study identified capabilities, opportunities, and motivations that can support older adults to transition from being a participating walker to becoming a volunteer walk leader. This complements research on volunteer motivation for a health walk programme in Australia [66] using self-determination theory [67,68].

#### 4.1.4. How Does the Chain of Antecedent and Emergent COM-Bs Affect Health and Well-Being among Older Adults with or at Risk of NCDs?

Participants largely came into the GOHW with poor estimations of their physical capabilities and acknowledgement of their recent or chronic health conditions, such as infection, falls, pain, recent operations, cardiovascular conditions, pulmonary conditions, neurological deficits, and depression. As they engaged with the GOHW and stayed with the programme, they improved their health and well-being by walking more, getting out in nature, and becoming leaders who maintained the walking group. Across the group, walkers identified that they were physically stronger, exercised more, lost weight, had fewer falls, were better able to cope with pain, and felt happier. Our qualitative examination of the social aspects of GOHW [28] identified the emergence of social well-being as well, including expanding social networks, meaningful relationships, a sense of belonging, and acting on empathy for others. For older adults with significant NCDs/chronic health problems, these outcomes added up to improved overall health, well-being, and quality of life. Our results are consistent with findings of improved health markers, health-related quality of life, and reduced depression in a systematic review of outdoor group walks that included 15 studies of older adults with a broad range of health conditions [40]. A recent ‘review of reviews’ investigating physical activity (broadly defined) found strong evidence from randomised controlled trials of improvement in both quality of life and well-being among older adults *without* chronic conditions [69]. This suggests a continuing need to include health outcomes in physical activity and nature-based intervention studies in older adults *with* chronic conditions.

### 4.2. Limitations and Strengths

Qualitative studies have several inherent limitations, including small sample size, limited socio-demographic diversity, and non-generalisability, issues which are of relevance to the study reported here. Ours was a purposive sampling of older adult walkers engaged in an activity tracker GOHW through which we sought to gain insight from their description of the experience. Although the participants were of limited ethnic diversity, this demographic reflects other UK studies of GOHWs [7]. The sample, however, did have a wide age range of older adults (50–80 years old) and a larger proportion of men than is typically reflective of GOHW [7]. To add context and understanding, one of the researchers twice accompanied the participants on a GOHW and to the after-walk social time. The material could be interpreted differently, and we did not check back with the participants to ensure our interpretations did not distort their experience. The data were, however, examined using both triangulation among researchers from different disciplines (environmental psychology, medicine, geography) and a search for disconfirming evidence related to our categorisations. Lastly, while findings clearly pertain to the individuals involved in the walking group being studied and are limited in transferability, the data provide rich experiential insights into COMs that facilitate joining a walking group, emerging COMs that change personal behaviours, including physical activity, and how this chain contributes to better health among older adults with NCDs. This person- and situation-specific richness is one of the strengths of qualitative research.

### 4.3. Future Research

Our study focused on the COMs’ relationship with behaviour. Given the hypothesised inter-relationships among the COMs themselves [11,51], future qualitative research could provide in-depth insights into these relationships within NBIs or NPIs. Our findings also identify a rich area of research into the processes of becoming a walk leader, which is an important aspect of ongoing group walking programmes.

The COM-B systems identified in this qualitative GOHW study could provide a basis for building validated scales to quantify the significance of various COMs that bring people into GOHW programmes. This approach could further elucidate emerging COMs that support adopting personal physical activity or more time in nature. Quantitative studies using these newly created COM scales, validated scales for motivation, reliable measures of physical activity, and appropriate measures of health outcomes would increase the utility and generalisability of our findings.

Future qualitative or quantitative research could use the COM-B model to compare the experiences of group participants who left the GOHW or who were not successful in establishing regular physical activity as well as those who remained involved. This comparison could provide further insight into how COMs interact with behaviours and support modification of programme/intervention designs.

Importantly, the use of the COM-B behaviour change system would benefit further investigations of other NBIs within other demographics and contexts. Additional studies would facilitate confidence in the utility of using the COM-B system in NBI research that pertains to the prevention and treatment of non-communicable health conditions.

### 4.4. Implications

The use of the COM-B system as a lens for identifying key drivers of engagement with a GOHW highlights how programme designs can be improved. Modifiable physical opportunities, such as offering a free activity tracker, and powerful social opportunities, like social norming when signposted from reliable health resources (e.g., national health systems), are influential for engagement. Automatic motivations can be enhanced by a programme design that offers safety and security. Personal resources (reflective motivations) such as a belief in the ability to change or an interest in goal setting could be supported through programmatic elements or other psychological resources, such as health coaching. Addressing negative COMs through programme design or additional resources could also remove barriers to engagement, such as poor physical capabilities due to ageing or health status, the lack of a mental map of outdoor places, and the habit of a sedentary lifestyle.

Providers of other NBIs, such as green gyms or outdoor exercise programmes, could also use our COM-B findings to both understand and improve their programmes. Critical to gaining benefits from nature are developing capabilities for nature engagement, such as expanded mental maps about the outdoor environment, including knowledge of paths, terrain, and weather. Attention to social norms related to the programme itself and social support for individuals within and outside the programme could enhance participation. Addressing psychological capabilities and motivations is an often neglected part of various NBIs. Adjusting activities in light of age or health status can help build physical capabilities and lead to successful independent activity. NBIs that utilize these approaches are more likely to serve the needs of their clients and support their health, well-being, and quality of life.

Currently, chronic health conditions and non-communicable diseases are the leading causes of death and poor quality of life. Policies that address risk factors, such as physical inactivity, by using programmes that connect people to nature have the potential to synergistically enhance both physical and mental health. Older adults in general enjoy walking as exercise and prefer walking with someone else, providing both social support and a sense of safety. Signposting or prescriptions from reliable health advocates with individualised knowledge of their client’s needs could systematically enhance the uptake of these programmes. Ultimately, NBIs, such as outdoor walking programmes, are low-cost to the health system, yet address a potent risk factor and provide both mental and physical health improvements.

## 5. Conclusions

We applied Michie et al.’s [11] COM-B behaviour change model to identify existing and emerging capabilities, opportunities, and motivations, their impact on behaviour, and on perceived physical and mental health among older adults in a nature-based intervention (NBI), a group outdoor health walk. To the best of our knowledge, this is the first application of the COM-B to a NBI for adults. The COM-B model allowed us to examine two separate behaviours, that of (i) engaging with the intervention itself, and (ii) incorporating the behaviour that the intervention targets. The analysis further identified emerging capabilities, opportunities, and motivations that supported additional health-promoting behaviours, including increased time outdoors in nature and leadership to self-organise continued group walks. This work offers insights into the design and promise of NBIs to effectively engage older adults with chronic health conditions and foster personal behaviour change for enhanced health and well-being.

## Figures and Tables

**Table 1:** Definitions for our application of COMs associated with the COM-B Model of Behaviour [11] for data analysis. COM-B—Capabilities, Opportunities, Motivations, and Behaviours. COMs—Capabilities, Opportunities, and Motivations.

COM-B Model Component Definitions		
COMs	Components	Definition
**Capability**	Physical	Determined by a person’s fitness or physical limitations
	Psychological	Determined by a person’s thought processes
**Opportunity**	Physical	Afforded by the physical environment
	Social	Afforded by the social and cultural settings
**Motivation**	Reflective	Driven by premeditated thoughts and plans
	Automatic	Driven by emotions and habits

## Data Availability

The (pseudonymized) data presented in this study are available on request from the corresponding author. The data are not publicly available to preserve the confidentiality of participants.
